# Ethylene Polymerization over Metal–Organic Framework-Supported Zirconocene Complexes

**DOI:** 10.1021/acscatal.4c01061

**Published:** 2024-05-29

**Authors:** Yaqi Wu, Joren M. Dorresteijn, Bert M. Weckhuysen

**Affiliations:** † Inorganic Chemistry and Catalysis group, Institute for Sustainable and Circular Chemistry and Debye Institute for Nanomaterials Science, Utrecht University, Universiteitsweg 99, 3584 CG Utrecht, The Netherlands; ‡ Hydrogen Energy Utilization and Energy Storage Technology Laboratory, Ningbo Institute of Materials Technology and Engineering, Chinese Academy of Sciences, Ningbo, Zhejiang 315201, P. R. China

**Keywords:** supported metallocene, ethylene polymerization, catalyst characterization, FT-IR spectroscopy, metal−organic frameworks

## Abstract

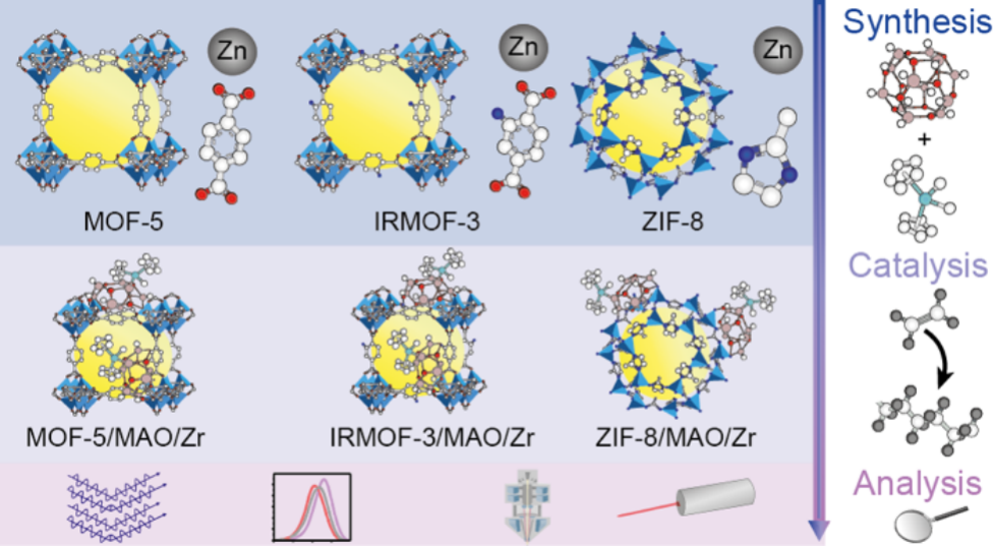

Metallocene immobilization onto a solid support helps to overcome the drawbacks of homogeneous metallocene complexes in the catalytic olefin polymerization. In this study, valuable insights have been obtained into the effects of pore size, linker composition, and surface groups of metal–organic frameworks (MOFs) on their role as support materials for metallocene-based ethylene polymerization catalysis. Three distinct Zn-based metal–organic frameworks (MOFs), namely, MOF-5, IRMOF-3, and ZIF-8, with different linkers have been activated with methylaluminoxane (MAO) and zirconocene complexes, followed by materials characterization and testing for ethylene polymerization. Characterization has been performed by multiple analytical tools, including X-ray diffraction (XRD), scanning electron microscopy (SEM), gel permeation chromatography (GPC), differential scanning calorimetry (DSC), and CO Fourier transform infrared (FT-IR) spectroscopy. It was found that the interactions between MOFs, MAO, and the zirconocene complex not only lead to both catalyst activation and deactivation but also result in the creation of multiple active sites. By alteration of the MOF support, it is possible to obtain polyethylene with different properties. Notably, ultrahigh molecular weight polyethylene (UHMWPE, *M*_W_ = 5.34 × 10^6^) was obtained using IRMOF-3 as support. This study reveals the potential of MOF materials as tunable porous supports for metallocene catalysts active in ethylene polymerization.

## 1 Introduction

With the multibillion polyolefin market ever increasing,^1,2^ it is clear that polyolefins are still used as the primary polymers for packaging, additives, and other consumer products.^3,4^ Their wide demand can be ascribed to their cost, stability, and broad applicability.^5,6^ Therefore, great effort has been continuously made over the decades by researchers to optimize productivity and property control for the different olefin polymerization processes. Kaminsky and Sinn’s discovery of metallocene complexes in the mid-1970s was thus a major advancement for the field.^7−9^ This discovery has led to increased possibilities in terms of tacticity control, property control, and incorporation of comonomers.^10^

Metallocenes (Cp_2_ML_2_) are a catalyst family comprising of group 4 transition-metal complexes bound to two cyclopentadienyl(-derivative) rings and two alternative ligands, mostly halides or alkyls.^11^ These single metal site catalyst materials usually exhibit higher catalytic activity than their predecessors, namely, the Ziegler–Natta catalyst materials (e.g., TiCl_4_ on MgCl_2_) and the Phillips catalyst materials (e.g., CrO_*x*_ on SiO_2_).^12^ This higher activity can be also attributed to the discovery of methylaluminoxane (MAO), a cocatalyst that greatly enhances the productivity of metallocene-based catalyst materials.^13^ This is, however, not the only function MAO has, as it greatly reduces the leaching of the metallocene complex from the surface when heterogenized on a support.^14^ For the utilization of metallocene complexes in industrial olefin polymerization applications, it needs to be heterogenized on a support for processability and for controlling the polymerization degree. Otherwise, this leads to uncontrolled olefin polymerization and results in reactor fouling, a very costly process.^15^ Generally, metallocene complexes are supported on a widely available inorganic support, such as SiO_2_. This support then acts as a template for controlled polymer growth and therefore the properties of the support influence the polymerization process significantly.^16^ Physical properties, such as surface area, porosity, particle size, and morphology, determine the polymer growth.^17−19^ Additionally, the chemical nature of the support material, not only limited to properties, such as hydroxyl content, friability, and acidity, also determines the effectivity of the catalyst material.^20−22^ Based on all of these previous observations, it can be established that optimizing the support material for making metallocene-based catalyst materials is as a result detrimental to catalytic olefin polymerization.

Metal–organic frameworks (MOFs) could possibly fulfill this role due to their high tunability of physical and chemical properties. MOFs are crystalline materials composed of inorganic nodes based on metal ions and their clusters, chained together by organic linkers. This results in virtually limitless potential regarding composition, functionality, and porosity.^23,24^ So far, MOFs have been utilized as materials for carbon capturing and storage (CCS), as well as in other applications, such as chemical sensing, gas adsorption, and separation.^25−27^ In the past decade, there has also been research on the use of MOFs for olefin polymerization catalysis. Numerous studies have already encompassed the effects of properties, such as porosity, active site, crystallite size, and organic linker on ethylene polymerization activity, and also produced polyethylene materials.^28−33^ However, comprehensive studies are still relatively rare for MOFs as support materials for metallocene complexes for catalytic olefin polymerization.

In this study, three Zn-based MOFs (i.e., MOF-5, IRMOF-3, and ZIF-8) are investigated as support for the metallocene complex Cp_2_ZrMe_2_ (bis(cyclopentadienyl)dimethyl-zirconium(IV)). We present data in which the organic linker has been varied to investigate the influence of pore structure, functional groups, and surface properties of MOFs as support material on catalytic ethylene polymerization. It is important to note that MOF-5 (Zn_4_O(BDC)_3_, BDC = 1,4-benzenedicarboxylate) and IRMOF-3 (Zn_4_O(NH_2_–BDC)_3_, NH_2_–BDC = 2-amino-1,4-benzenedicarboxylate) with the organic linker of BDC and NH_2_–BDC exhibit the same framework structure, but a different pore size of 12 and 15 Å in diameter, respectively.^34−36^ On the other hand, ZIF-8 (Zn(MeIM)_2_, MeIM = 2-methylimidazole) with the linker of MeIM possesses a pore size of 11.6 Å in diameter, with the pores yet connected by small apertures of 3.4 Å.^37^

## 2 Experimental Section

### 2.1 Catalysts Synthesis

*Supported-catalysts preparation*: All synthetic procedures were carried out in a N_2_ glovebox, and all of the samples were also stored under an inert N_2_ atmosphere in a well-sealed glass vial covered by aluminum foil. All of the solvents, including toluene and *n*-pentane, used for the synthesis were degassed by N_2_ bubbling and dried by molecular sieves over the weekend to remove water. The MOF-supported metallocene catalysts were prepared following a 2-step procedure: MAO impregnation and metallocene impregnation. *MAO impregnation*: 0.5 g of MOF was dispersed into 20 mL of toluene to form a slurry. Then, a solution of 16% MAO was added into the MOF/toluene slurry under reflux and gentle stirring at 125 °C for 4 h. Then the products were washed 3 times with toluene and 3 times with *n*-pentane and dried under vacuum. The three as-prepared activators were named as MOF-5/MAO, ZIF-8/MAO, and IRMOF-3/MAO, respectively. *Zirconocene impregnation*: a determined amount of metallocene catalyst Cp_2_ZrMe_2_ was added to the obtained MOF-supported MAO/toluene slurry under gentle stirring at room temperature for 2 h. After stirring, the products were washed twice with toluene and once with pentane. The obtained catalysts were named as MOF-5/MAO/Zr, ZIF-8/MAO/Zr, and IRMOF-3/MAO/Zr, respectively.

### 2.2 Catalyst Testing

Ethylene polymerization of MOF-supported zirconocene catalysts was performed in a slurry-phase Parr autoclave reactor at 10 bar and room temperature. For a typical ethylene polymerization experiment, 20 mg of supported catalyst, 15 mL of heptane solvent, and 0.25 mL of scavenger agent triethylaluminum (TEAL) in toluene were first added to a glass vial and then placed inside the autoclave reactor. Then the reactor was pressurized with ethylene gas (C_2_H_4_ 4.0, Linde) to 10 bar. During the whole polymerization reaction, the pressure of ethylene was kept at 10 bar and stirred at 600 rpm. The polymerization was terminated by turning off the ethylene feed. The polymer product was collected and washed with ethanol for 3 times and dried at 60 °C overnight.

### 2.3 Catalyst Characterization

X-ray diffraction (XRD) patterns were collected on a Bruker D2 Advance diffractometer in Bragg–Brentano geometry using Co Kα radiation (λ = 1.79026 Å) and Cu Kα radiation (λ = 1.540 Å) (polymers with Cu Kα), operating at 30 kV, respectively. Scanning electron microscopy (SEM) images were collected on a Phenom ProX and EI Helios NanoLab G3 UC, respectively. The Al, Zr, and Zn contents of the materials investigated were determined by inductively coupled plasma-optical emission spectroscopy (ICP-OES, PerkinElmer Optima 8300 Optical Emission Spectrometer and Thermo Fisher iCAP PRO). The specific surface area and pore volume of materials investigated were measured by a volumetric adsorption analyzer (Micromeritics TriStar 3000 and Micromeritics ASAP 2020) using nitrogen as adsorbate at −196 °C. The specific surface area (*S*_BET_) was determined by the Brunauer–Emmett–Teller (BET) method. Pore size distributions (PSDs) were obtained by nonlocal density functional theory (NLDFT). The samples were loaded inside a N_2_ glovebox, and no pretreatment was necessary prior to physisorption measurements. Differential scanning calorimetry (DSC) was carried out using a DSC 214 (NETZSCH, Germany) instrument. Two successive heating cycles and one cooling cycle were performed from room temperature to 200 °C and at a heating rate of 10 °C/min. Fourier transform infrared (FT-IR) spectroscopy was performed with CO as the probe molecule. Sample pellets 5 mm in diameter were prepared inside a N_2_ glovebox by pressing 5.2 mg of each material in a stainless-steel collar. Then the pellets were placed in a transmission IR cell fitted with CaF_2_ windows inside the glovebox. The well-closed IR cell was taken out of the glovebox and evacuated carefully for 30 min and cooled with liquid N_2_ to −188 °C. The sample was then dosed with increasing amounts of CO (10% in He, 99.9% purity) from 0.1 to 5 mbar. The FT-IR spectra were recorded with a PerkinElmer 2000 spectrometer with 32 scans and a resolution of 4 cm^–1^. The molecular weight *M_n_* (number-average molecular weight) and *M*_w_ (weight-average molecular weight), and molecular weight distributions D (*M*_w_/*M_n_*) and *D*′ (M_*z*_/*M*_w_) were determined by size exclusion chromatography (SEC) and in particular by IR-detected gel permeation chromatography (GPC) at a high temperature (145 °C) (for more details, see Supporting Information Additional experimental section).

## 3 Results and Discussion

**Figure 1:**
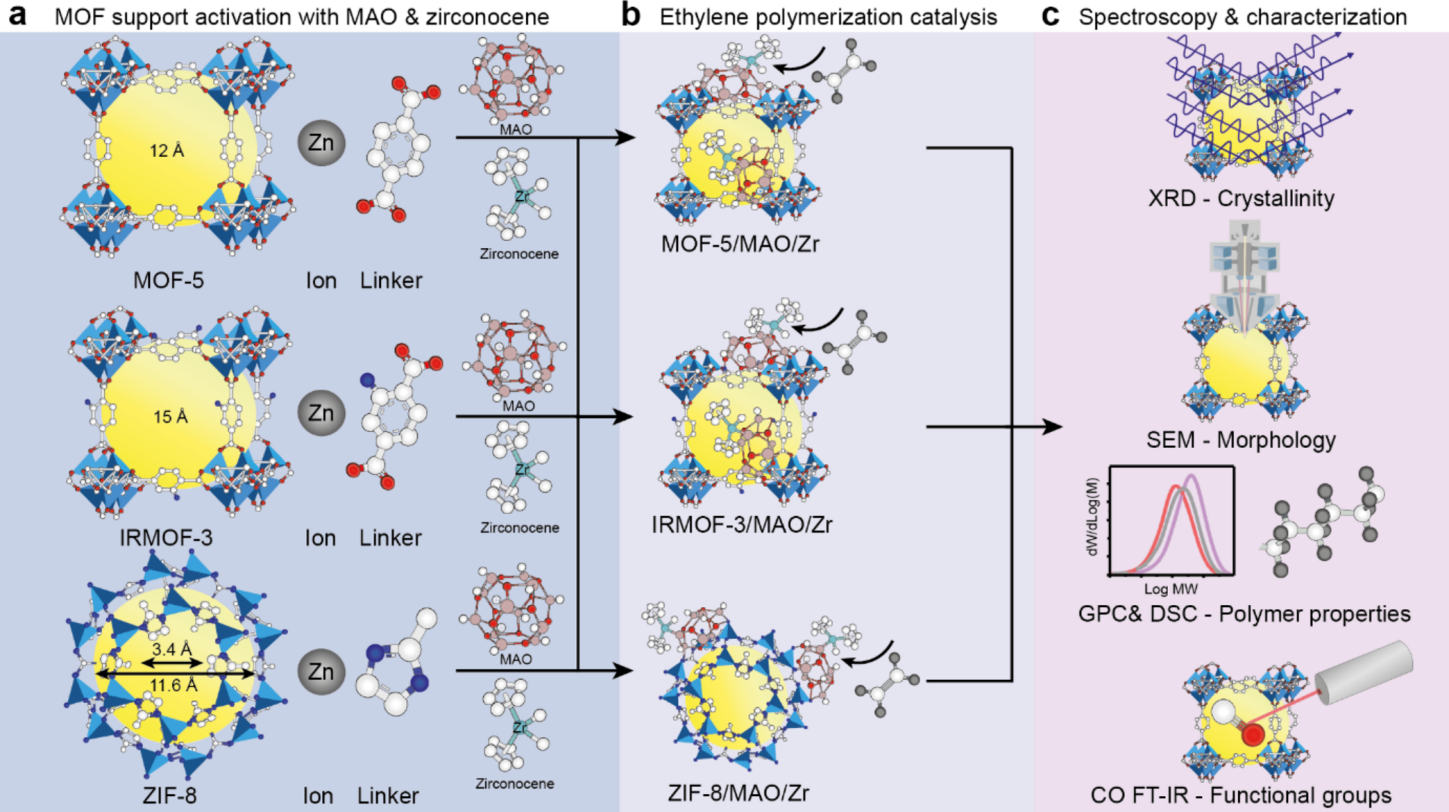
Schematic illustration of using three distinct Zn-based metal–organic frameworks (MOFs), namely, MOF-5, IRMOF-3, and ZIF-8, with different linkers as support for zirconocene-based ethylene polymerization. (a) MOF support activation with methylaluminoxane (MAO) and zirconocene complexes. (b) The synthesized MOF-supported zirconocene catalysts were used for ethylene polymerization. (c) Insights into MOFs as support materials in ethylene polymerization were gained by multiple analytical tools, including X-ray diffraction (XRD), scanning electron microscopy (SEM), gel permeation chromatography (GPC), differential scanning calorimetry (DSC), and CO Fourier transform infrared (FT-IR) spectroscopy.

We prepared supported MAO by the impregnation of the MOF support with a 16% MAO solution. Subsequently, the catalyst materials were prepared by the loading of this MOF-supported MAO with the metallocene Cp_2_ZrMe_2_ complex. The microstructures, chemical properties, and morphologies of a set of MOF-supported MAO (MOF/MAO) and corresponding catalysts (MOF/MAO/Zr) are subsequently characterized by X-ray diffraction (XRD), N_2_ physisorption, inductively coupled plasma-optical emission spectroscopy (ICP-OES), and scanning electron microscopy (SEM). The performance in catalytic ethylene polymerization of the various MOF-supported catalyst materials and the properties of the obtained polyethylene (PE) are also studied. The surface properties of the MOF, MOF/MAO, and MOF/MAO/Zr materials are investigated with Fourier transform infrared spectroscopy (FT-IR) with CO as the probe molecule. The results obtained provide valuable insights into the surface chemical groups and pore size of MOFs as support materials for metallocene. These parameters have a significant impact on the impregnation and activation of MAO and the zirconocene complex, which also explains the different catalytic performance of these MOF-supported catalyst materials in catalytic ethylene polymerization. The approach is schematically shown in Figure 1. In what follows, we will discuss first the characterization data of the different catalyst materials, thereby focusing on the effect of the MAO and zirconocene loading on the generation of the active sites. The second part describes the ethylene polymerization data of these materials, including a detailed analysis of the polyethylene made.

### 3.1 Catalyst Characterization and the Formation of Active Sites

**Figure 2:**
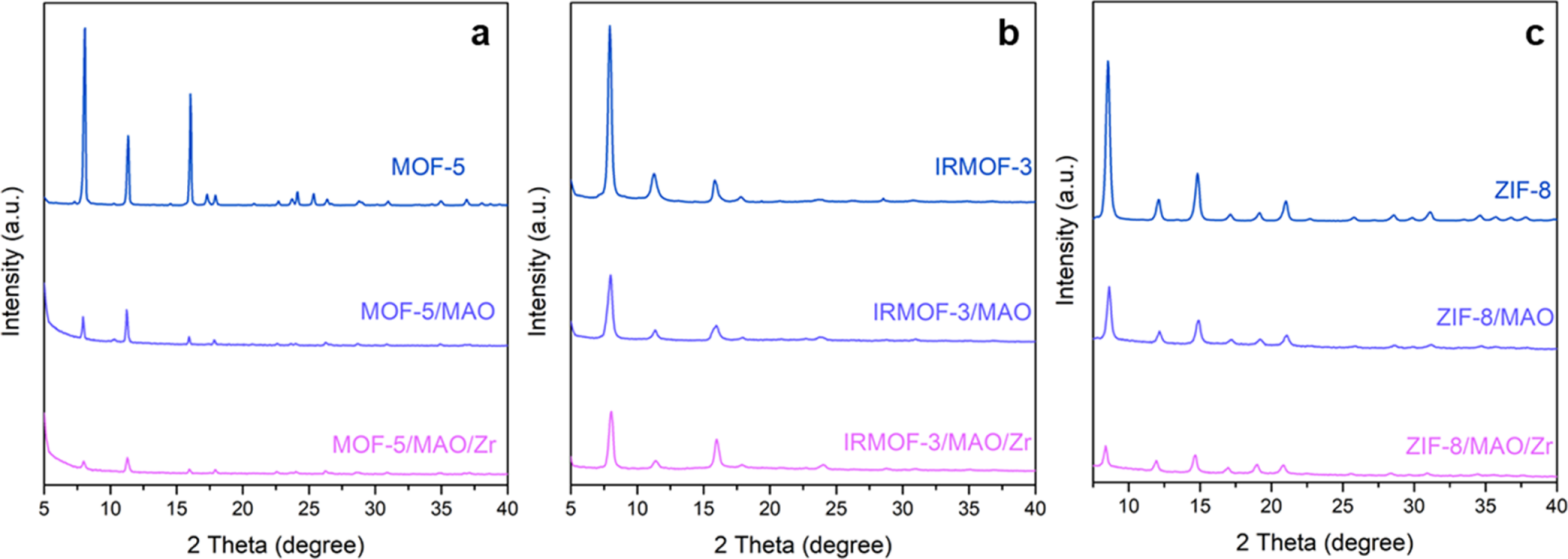
X-ray diffraction (XRD) patterns of (a) MOF-5, (b) IRMOF-3, and (c) ZIF-8 before and after methyaluminoxane (MAO) impregnation and zirconocene loading.

The properties of the pristine MOFs and MOF-supported catalyst materials have been studied by a variety of analytical methods. The successful syntheses of MOF-5, IRMOF-3, and ZIF-8 are confirmed by the XRD patterns (Figure 2). The crystallinity of all of the investigated MOFs progressively decreases upon MAO impregnation and subsequent zirconocene loading. It is important to note that the MOF-5 material is the most affected (the crystallinity decreased from 82.2 to 29.2%), followed by the ZIF-8 material (the crystallinity decreased from 82.9 to 56.5%) and the IRMOF-3 material (the crystallinity decreased from 81.6 to 65.1%), particularly after zirconocene immobilization (Figure S1). These observations suggest that the interactions between MOFs, MAO, and the zirconocene complexes are noninnocent and most probably lead to a partial destruction of the crystalline structure of the MOF materials under study.

**Figure 3:**
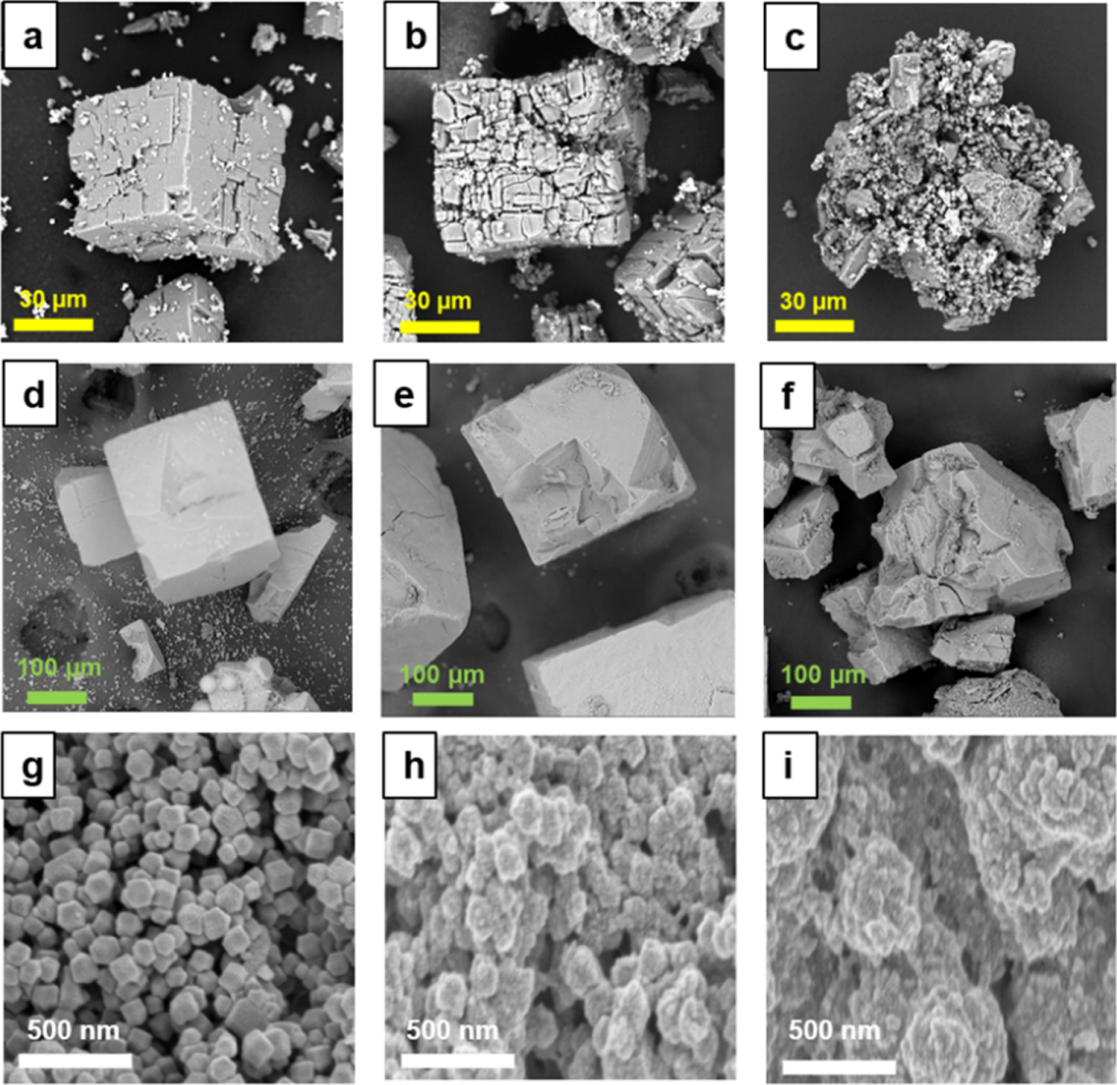
Scanning electron microscopy (SEM) images of (a) MOF-5, (b) MOF-5/MAO, (c) MOF-5/MAO/Zr, (d) RMOF-3, (e) IRMOF-3/MAO, (f) IRMOF-3/MAO/Zr, (g) ZIF-8, (h) ZIF-8/MAO, and (i) ZIF-8/MAO/Zr.

The SEM results, shown in Figure 3, confirm this hypothesis, as one can note the direct observation of MOF particle damage after MAO impregnation and zirconocene loading. It is evident that the three pristine MOFs are fully intact, with MOF-5 and IRMOF-3 appearing as cubic crystals, while ZIF-8 consists of rhombic dodecahedron-shaped crystals. After MAO impregnation, cracks can be observed on the surface of the MOF-5 crystals, while the IRMOF-3 crystals show no visible changes, and the originally smooth surface of the ZIF-8 crystals is covered with a rough layer. After loading the zirconocene complexes, the MOF-5 crystals are broken up into even smaller pieces, while crystal breakage is also observed for IRMOF-3. In the case of ZIF-8 crystals, their surfaces become fuzzier. Hence, the SEM results are consistent with the XRD results that after MAO and zirconocene loading, the MOF crystals undergo crystal breakage and surface damage, leading to a significant decrease in their crystallinity.

**Figure 4:**
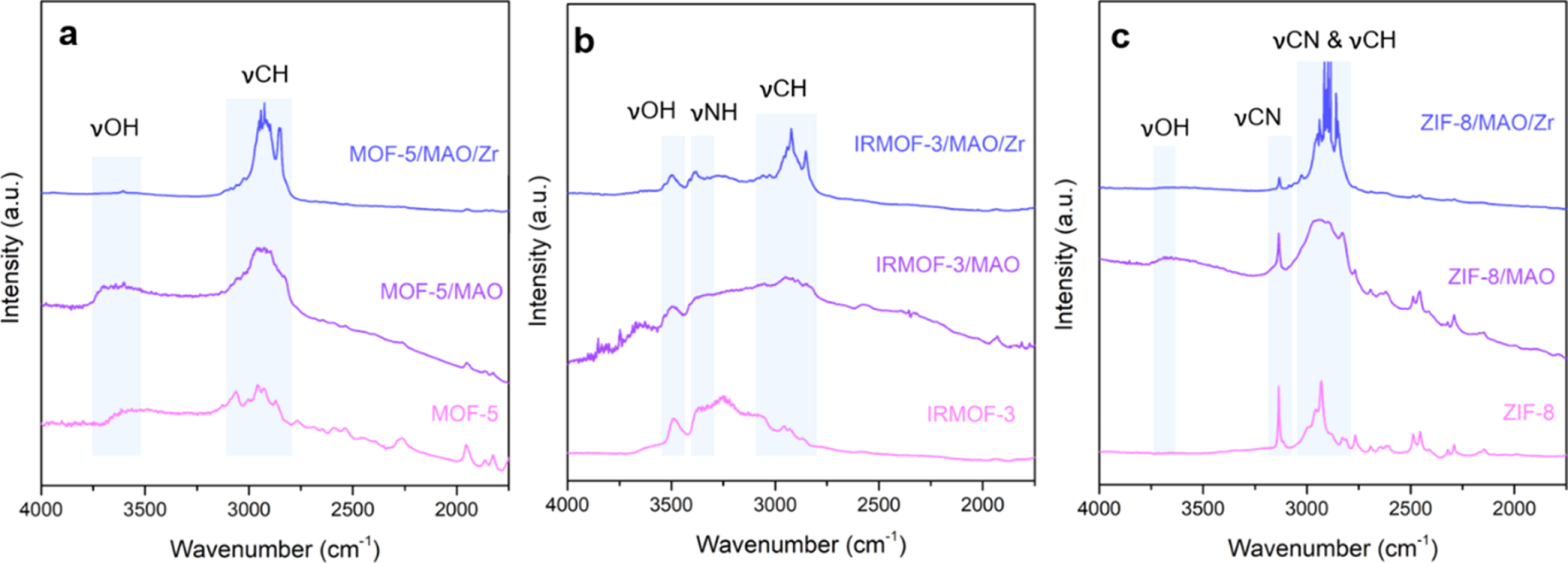
Fourier transform infrared (FT-IR) spectra of (a) MOF-5, (b) IRMOF-3, and (c) ZIF-8 before and after methylaluminoxane (MAO) and zirconocene loading and measured under vacuum conditions.

The FT-IR spectra of the different MOFs and MOF-supported catalyst materials under a vacuum are shown in Figure 4. The chemical structures of these MOFs are affected due to the loading of MAO and zirconocene, with certain characteristic vibrational peaks of the MOFs decreasing in intensity or even completely disappearing. For the MOF-5 and IRMOF-3 crystals, the νOH of hydroxyl groups (visible in the 3600–3300 cm^–1^ region) located on the external surfaces/at internal defects and the νCH of the aromatic rings (visible at ∼3066 cm^–1^) were observed.^38^ For the ZIF-8 crystals, aliphatic and aromatic νCN and νCH vibrations were observed. After MAO impregnation, additional vibrational peaks at ∼2960–2880 cm^–1^ can be ascribed to the CH_3_ vibrations, indicating the successful grafting of MAO onto the MOF materials. After loading these MOF/MAO materials with zirconocene complexes, in addition to the peaks of the CH_3_ vibrations centered at ∼2960–2880 cm^–1^, a vibration at ∼2854 cm^–1^ can be ascribed to the self-condensation of MAO.^39^ Furthermore, vibrations assigned to perturbed νOH were observed for the three MOF materials after MAO impregnation, while νOH (∼3490 cm^–1^) and νNH (∼3388 cm^–1^) vibrations for all IRMOF-3 samples under study indicated the remaining NH_2_ groups and OH groups of the MOF materials after MAO and zirconocene loading.

In Table 1, the elemental analysis results, as obtained by inductively coupled plasma-optical emission spectroscopy (ICP-OES), are summarized. These results reveal that MAO and the zirconocene complexes are successfully grafted onto the MOF-5, IRMOF-3, and ZIF-8 materials with different loading efficiencies and hence amounts of Al and Zr. Al amounts of 19.3, 6.86, and 22.5 wt % have been obtained for MOF-5/MAO, IRMOF-3/MAO, and ZIF-8/MAO, respectively. After zirconocene loading, Zr amounts of 2.57, 0.16, and 0.55 wt % and Al/Zr ratios of 23, 91, and 101 were obtained for MOF-5-, IRMOF-3-, and ZIF-8-supported catalysts, respectively. These three Zn-based MOF materials exhibited quite different MAO and zirconocene loading efficiencies and also a lower Al/Zr ratio, compared with silica-supported metallocene complexes.^14^

**Table 1:** Elemental Analysis and Porosities of Metal–Organic Frameworks (MOFs) after Methylaluminoxane (MAO) Impregnation and Zirconocene Loading, as Obtained by Inductively Coupled Plasma-Optical Emission Spectroscopy (ICP-OES)

sample		Al (wt %)	Zr (wt %)	Al/Zr molar ratio	*S*_BET_ (m^2^/g)	pore volume (cm^3^/g)
Zn-MOFs	MOF-5				551	0.28
	IRMOF-3				382	0.24
	ZIF-8				1290	1.03
MAO impregnation	MOF-5/MAO	19.3			132	0.05
	IRMOF-3/MAO	6.86			221	0.11
	ZIF-8/MAO	22.5			626	0.26
zirconocene loading	MOF-5/MAO/Zr	17.9	2.57	23	64	0.02
	IRMOF-3/MAO/Zr	4.34	0.16	91	46	0.02
	ZIF-8/MAO/Zr	16.5	0.55	101	124	0.05

It is important to mention here that the porosity parameters of these MOF materials after MAO impregnation and zirconocene loading provide an explanation for the different Al and Zr amounts achieved. MAO and zirconocene could enter inside MOF-5 because of its large pore size, exhibiting high Al and Zr amounts and a low surface area of the MOF-5/MAO and MOF-5/MAO/Zr materials (i.e., ∼132 and ∼54 m^2^/g, respectively). For IRMOF-3, the amino functional group on its linker would prevent the proper access of MAO and zirconocene complexes, resulting in lower amounts of Al and Zr and decent surface areas of the IRMOF-3/MAO and IRMOF-3/MAO/Zr materials (i.e., ∼221 and ∼46 m^2^/g, respectively). While the remaining relatively higher surface area of ZIF-8/MAO and ZIF-8/MAO/Zr (i.e., ∼626 and ∼124 m^2^/g) is likely due to the small aperture (3.4 Å) of the ZIF-8 material, which limits the entrance of the large MAO and zirconocene complexes, which is for MAO under reflux conditions around 19 Å.^40^ Hence, the MAO and zirconocene complexes might just mainly be grafted onto the surface of ZIF-8.

**Figure 5:**
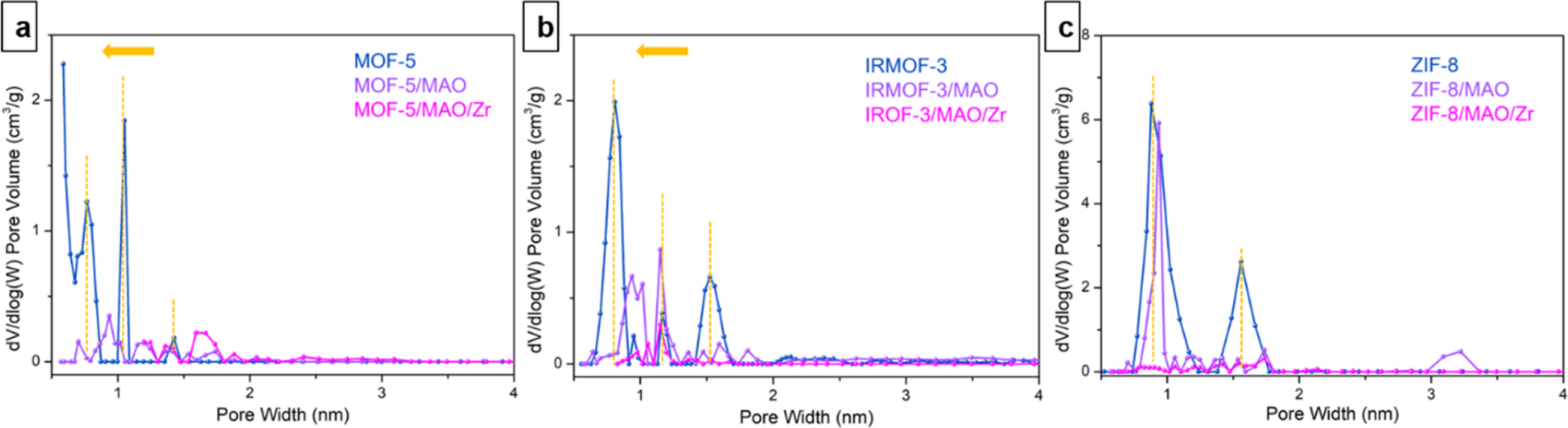
Nonlocal density functional theory (NLDFT) pore size distributions of (a) MOF-5, (b) IRMOF-3, and (c) ZIF-8 before and after methylaluminoxane (MAO) and zirconocene loading.

The NLDFT pore size distribution analysis is further performed to support the loading details of MAO and zirconocene. As shown in Figure 5, the data clearly illustrate the changes in pore size among pristine MOFs, MOF/MAO, and MOF/MAO/Zr samples. After MAO loading, MAO/MOF-5 and MAO/IRMOF-3 present a smaller pore width than the pristine materials, indicating that MAO is mainly loaded inside the particles. The PSD change between the pristine and MAO-loaded IRMOF-3 is less pronounced than that of MOF-5, which may be due to its much lower MAO loading amount. For ZIF-8, after MAO loading, the dominant peak at ∼0.9 nm shows no significant change. The one at ∼1.6 nm is affected, and we speculate that this may be due to the abundant surface groups of ZIF-8, leading to a high loading amount of MAO and causing a partial disruption to the ZIF-8 framework. By further combining the SEM images (Figure 3g–i), PSDs, and intrinsic pore structure characteristic of ZIF-8, we can infer that the MAO and zirconocene complexes are mainly grafted on the outside of the ZIF-8 particle. After Zr loading, the surface areas and crystallinity of these MOFs are further decreased, leading to less sharpness and less regularity in the pore size distribution. Overall, the MAO and zirconocene complexes are successfully grafted into MOF-5, IRMOF-3, and ZIF-8 in different degrees, as confirmed by FT-IR spectroscopy, elemental results, and porosity analysis. Moreover, the immobilization of MAO and zirconocene complexes resulted in a decrease in the crystallinity and also a partial destruction of the MOF crystals, including morphology changes.

### 3.2 Ethylene Polymerization Performance and Related Polyethylene Properties

The ethylene polymerization performance of the three distinct MOF-supported metallocene catalyst materials under study is summarized in Table 2. All MOF-supported catalyst materials show good ethylene polymerization activity. Among them, the MOF-5/MAO/Zr material performed the best with an activity of 373 kg of PE (mol Zr)^−1^ h^–1^, followed by 293 kg of PE (mol Zr)^−1^ h^–1^ for the IRMOF-3/MAO/Zr material and 269 kg of PE (mol Zr)^−1^ h^–1^ for the ZIF-8/MAO/Zr material. The high activity of the MOF-5/MAO/Zr material can be explained by the large pore size of MOF-5 and the high loading efficiency of both MAO and zirconocene (Al/Zr ratio of 23). However, the high loading of MAO and zirconocene also leads to the dramatic decrease of crystallinity and specific surface area in the MOF-5 material, thereby limiting the diffusion of ethylene molecules and exhibiting a moderate catalytic performance. Although IRMOF-3 also possesses large pores, the loadings of both MAO and metallocene are relatively low and the Al/Zr ratio is high of 91. This is probably because the amino groups on its linker molecule can act as poisonous Brønsted acid sites that can interact with MAO and therefore decrease the loading of the zirconocene complex, thereby leading to a high Al/Zr ratio. We speculate that this may also explain why IRMOF-3/MAO/Zr exhibits a high catalytic activity and also an ultrahigh molecular weight polyethylene (UHMWPE) product was obtained. In the case of the ZIF-8 material, the small ring aperture would limit the entry of MAO and zirconocene. Moreover, the presence of various surface groups on the surface of ZIF-8 facilitates the efficient grafting of MAO, yet some of them would also cause deactivation of the zirconocene complexes, leading to an Al/Zr ratio of 101 and a low ethylene polymerization catalytic activity. For these three MOF-supported catalyst materials, the catalytic activity is higher with a lower Al/Zr ratio, which is different for silica-supported metallocene catalyst materials, indicating there may be a decrease in the required amount of MAO for MOF-supported metallocene complexes.

**Table 2:** Catalytic Activity of the Three Metal–Organic Framework (MOF)-Supported Metallocene Catalyst Materials under Study and the Physicochemical Properties of the Polyethylene (PE) Products Obtained from the Ethylene Polymerization Experiments Performed

catalyst		MOF-5/MAO/Zr	IRMOF-3/MAO/Zr	ZIF-8/MAO/Zr
	activity^a^	373	293	269
	*M*_W_ (×10^6^)^b^	3.05	5.34	2.17
	*M*_N_ (×10^6^)^b^	0.54	0.99	0.49
	PDI	5.7	5.4	4.5
	*T*_c_ (°C)	114	117	118
	*T*_m_ (°C)^(I)^	142	140	138
1st cycle	Δ*H*_f_ (J/g)^(I)^	201	208^d^	205
	%*X*_d_^(I)^^c^	201	72^d^	71
	*T*_m_ (°C)^(II)^	139	135	136
2nd cycle	Δ*H*_f_ (J/g)^(II)^	156	150^d^	172
	%X_d_^(II)^^c^	54	52^d^	59

a Activity: kg PE (mol Zr)^−1^ h^–1^.

b Weight-average molecular weight (*M*_W_), number-average molecular weight (*M*_N_), and polydispersity index (PDI) determined by gel permeation chromatography (GPC) calibrated with narrow standards of polystyrene (PS).

c In comparison to melting heat of a 100% crystalline PE (290 J/g).

d This value is the crystallinity after correcting for the significant catalyst residue contribution in the final PE product.

**Figure 6:**
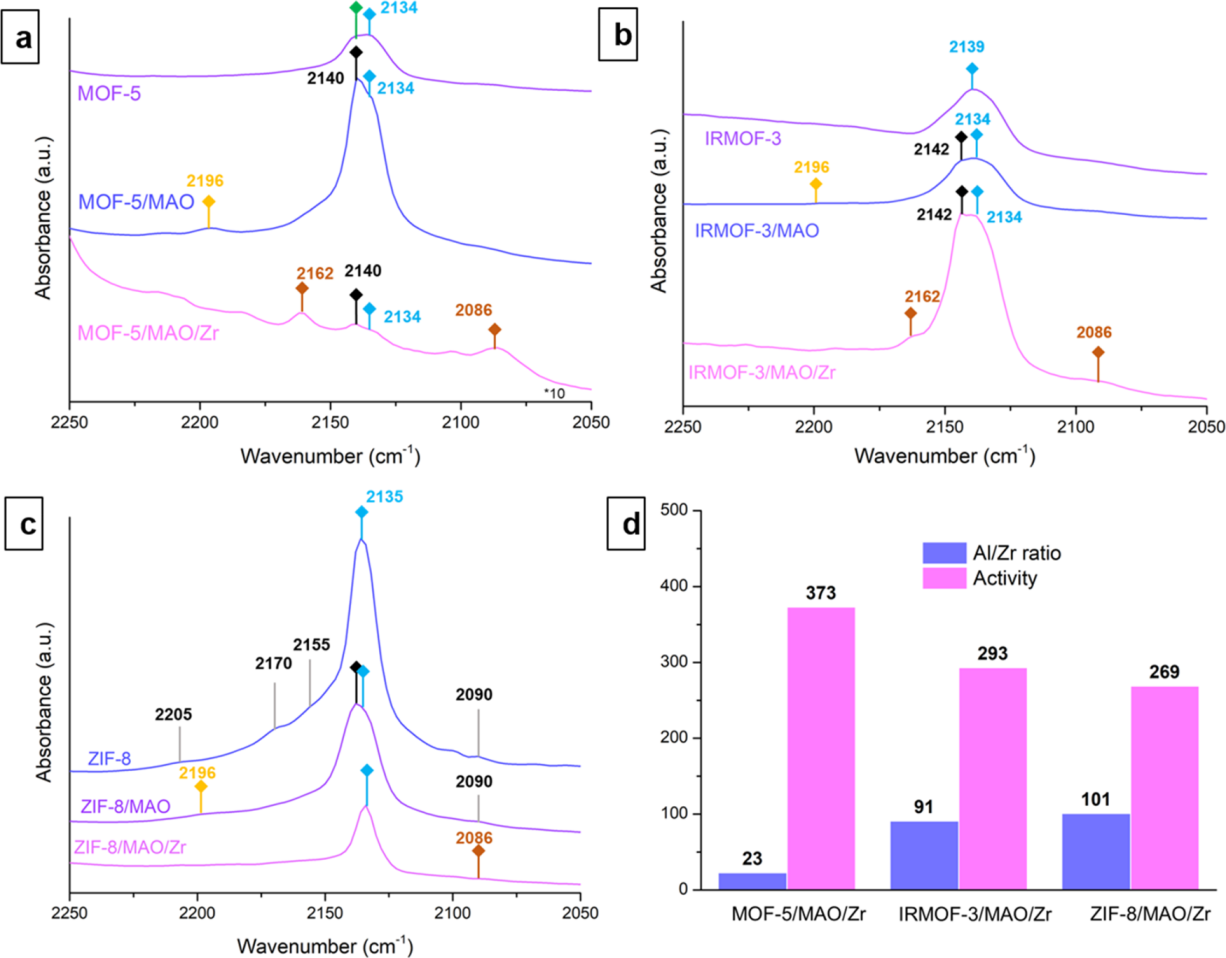
Fourier transform infrared (FT-IR) spectra for (a) MOF-5, (b) IRMOF-3, and (c) ZIF-8 before and after methylaluminoxane (MAO) impregnation and zirconocene loading upon CO adsorption at −188 °C and 1 mbar. (d) Histogram of Al/Zr ratio and ethylene polymerization catalytic activity of MOF-5/MAO/Zr, IRMOF-3/MAO/Zr, and ZIF-8/MAO/Zr. Bands: CO adsorbed on O^2–^ on the MAO surface at 2140–2142 cm^–1^ (black ◆), on weak Lewis acid sites at 2196 cm^–1^ (yellow ◆), physisorbed CO at 2136 cm^–1^ (blue ◆), on Zr cationic species at 2162 and 2086 cm^–1^ (brown ◆), on NH groups at 2170 and 2155 cm^–1^, on Zn^2+^ sites at 2205 cm^–1^, on OH groups and N^–^ moieties at 2090 cm^–1^, and CO molecules slightly perturbed through the carbon end or the oxygen end of MOF-5 at 2140 (green ◆).

Additional FT-IR spectroscopy measurements with CO as the probe molecule were performed to investigate the influence of surface properties of MOF supports on their ethylene polymerization performances. Figure 6 reports the FT-IR spectra of MOFs, MOF-supported MAO, and MOF-supported catalyst materials after CO adsorption at a pressure of 1 mbar. Moreover, the FT-IR spectra with increasing CO pressure for all samples under study are shown in Figure S2, allowing for the observation of clearer characteristic peaks of adsorption sites. The presence of Lewis acid sites derived from the Al centers of the MAO cocatalyst is confirmed with the appearance of new peaks at ∼2196 cm^–1^ for these MOFs after MAO impregnation.^14^ The additional vibrational peaks ascribed to CO adsorbed on Zr cationic species at ∼2162 and ∼2086 cm^–1^ are also observed for all MOF-supported Zr catalysts.^14^ The adsorption of CO on MOF-5 results in two main vibrational bands, located at ∼2134 and ∼2140 cm^–1^, corresponding to physisorbed CO and CO molecules slightly perturbed through the carbon end or the oxygen end of MOF-5, respectively.^38^ For the MOF-5/MAO material, the high loading amount of MAO on MOF-5 leads to a decrease in the crystallinity and surface area of MOF-5. As a result, the sharp vibrational band observed at ∼2140 cm^–1^ can be primarily ascribed to the CO interacting with the O^2–^ on the MAO surface rather than perturbed CO molecules.

It was found that the acidity of the support plays an important role in metallocene-based olefin polymerization catalyst materials. The Lewis acid sites would cause active sites for olefin polymerization, while weak Brønsted acid sites, such as OH groups and NH groups, would cause inactive species for catalytic olefin polymerization.^39,41−46^ The amino groups on the linker of IRMOF-3 can act as a poisonous Brønsted acid site that can interact with MAO. This is probably why vibrational bands assigned to physisorbed CO and CO adsorbed on O^2–^ on the MAO surface at ∼2134 and ∼2142 cm^–1^ were still observed for the IRMOF-3/MAO/Zr material. The FT-IR spectra of ZIF-8 after CO adsorption (Figure 6c) confirmed the presence of Brønsted acid sites, Lewis acid sites, and basic sites, with the vibrational bands at ∼2170 and ∼2155 cm^–1^ for CO adsorbed on NH Groups, a vibrational band at ∼2205 cm^–1^ for CO adsorbed on Zn^2+^ sites, and a vibrational band at ∼2205 cm^–1^ for CO adsorbed on OH groups and N^–^ moieties.^47^ Therefore, we speculate that the complicated surface groups, the N^–^ moieties, and OH groups in particular, of ZIF-8 result in the deactivation of the metallocene complexes, leading to a relatively low Zr content and a low ethylene polymerization activity despite having a relatively high Al content. In general, the surface groups of the MOF materials have a significant impact on the role of MOF as a catalyst support, and some surface groups can lead to the deactivation of metal catalyst materials. Therefore, when choosing MOF materials as catalyst supports for metallocene-based catalyst materials, it is necessary to take their surface groups into consideration.

**Figure 7:**
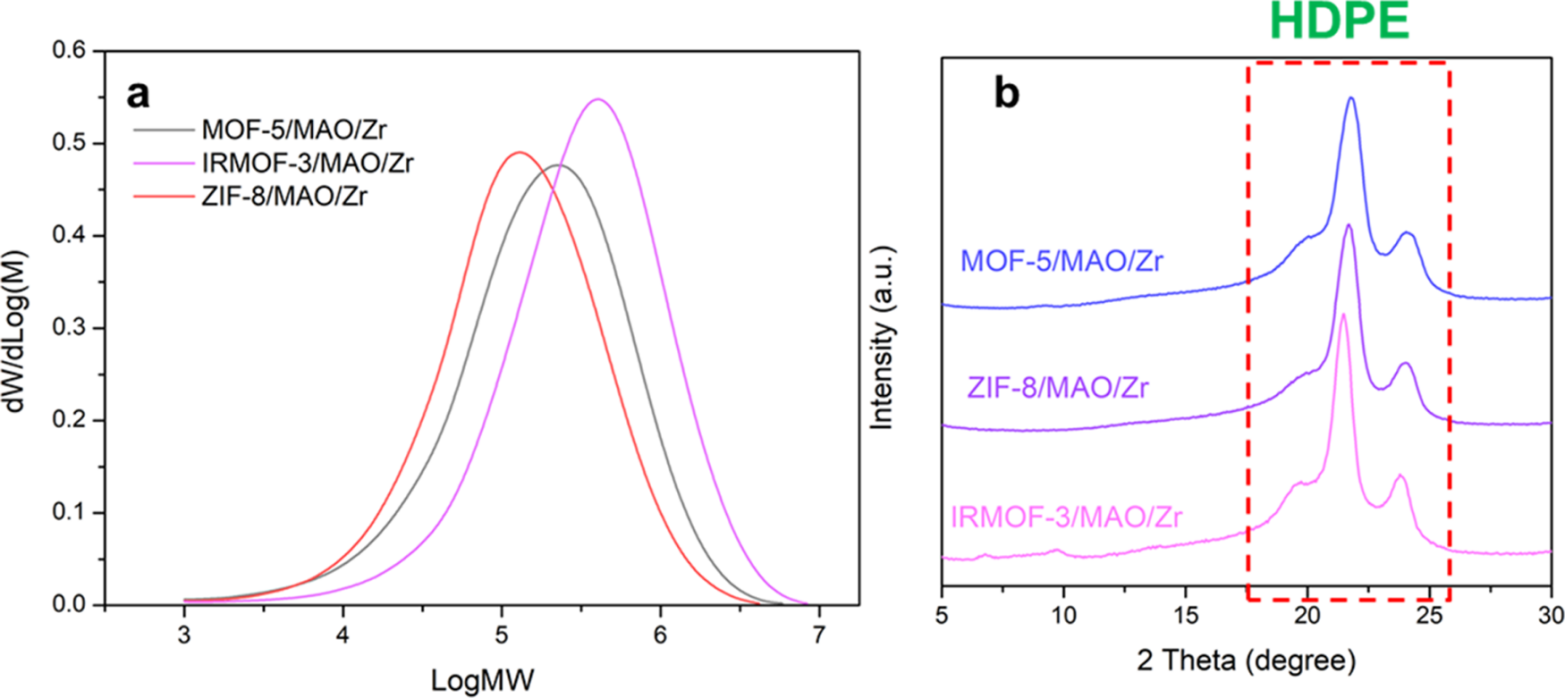
(a) Gel permeation chromatography (GPC) results and (b) X-ray diffraction (XRD) patterns of polyethylene (PE) produced from the metal–organic framework (MOF)-supported metallocene catalyst materials.

In the next step of our study, we have characterized the properties of PE made by the different catalyst materials. The gel permeation chromatography (GPC) results of PE made by the MOF-supported metallocene-based catalyst materials are shown in Table 2. Table 2 summarizes weight-average molecular weight (*M*_W_) of 3.05 × 10^6^, 5.34 × 10^6^, and 2.17 × 10^6^ for PE, as produced by the MOF-5-, IRMOF-3-, and ZIF-8-supported catalysts, respectively. It is notable that the *M*_w_ of PE from IRMOF-3-supported catalyst (5.34 × 10^6^) is a characteristic of ultrahigh molecular weight polyethylene (UHMWPE).^48,49^Figure 7a shows the GPC curves and molecular weight distributions (MWDs) of PE produced by the three catalyst materials under study. The polydispersity index (PDI) of the polymers produced by the MOF-5-, IRMOF-3-, and ZIF-8-supported catalyst materials are 5.7, 5.4, and 4.5, respectively. As we know that the polyolefins made with single-site catalyst materials, such as silica-supported metallocenes, have a PDI value close to 2, while the polyolefins produced by a catalyst containing more than one active site type, such as Ziegler–Natta and Phillips catalysts, have PDI range from 3 to 10, or even up to 20.^4,50,51^ Interestingly, the PE produced with the MOF-supported zirconocene complexes, as described in this study, exhibited PDI values located within the PDI range typically for PE produced by multisite olefin polymerization catalyst materials rather than by single-site olefin polymerization catalyst materials. It is hypothesized that the interactions between the MOF materials and the zirconocene complexes result in changes in the single-site nature of the metallocene complex so that the MOF-supported metallocene catalyst materials no longer exhibit single-site behavior.

We also performed differential scanning calorimetry (DSC) analysis on the polymers made to study the thermal behavior and to compare the properties of the produced PE products. Melting temperature (*T*_m_), crystallization temperatures (*T*_c_) with corresponding enthalpies of fusion (Δ*H*_f_), and crystallinity *X*_d_ of PE products by the three MOF-supported catalysts are obtained by DSC analysis, giving information about the molecular structure and density of the semicrystalline PE polymer.^52^ As shown in Table 2, the three PE products exhibit melting temperatures of 139, 135, and 136 °C and crystallinities of 52–59%, showing typical ranges of high-density polyethylene (HDPE) with a low branching degree.^52,53^

**Figure 8:**
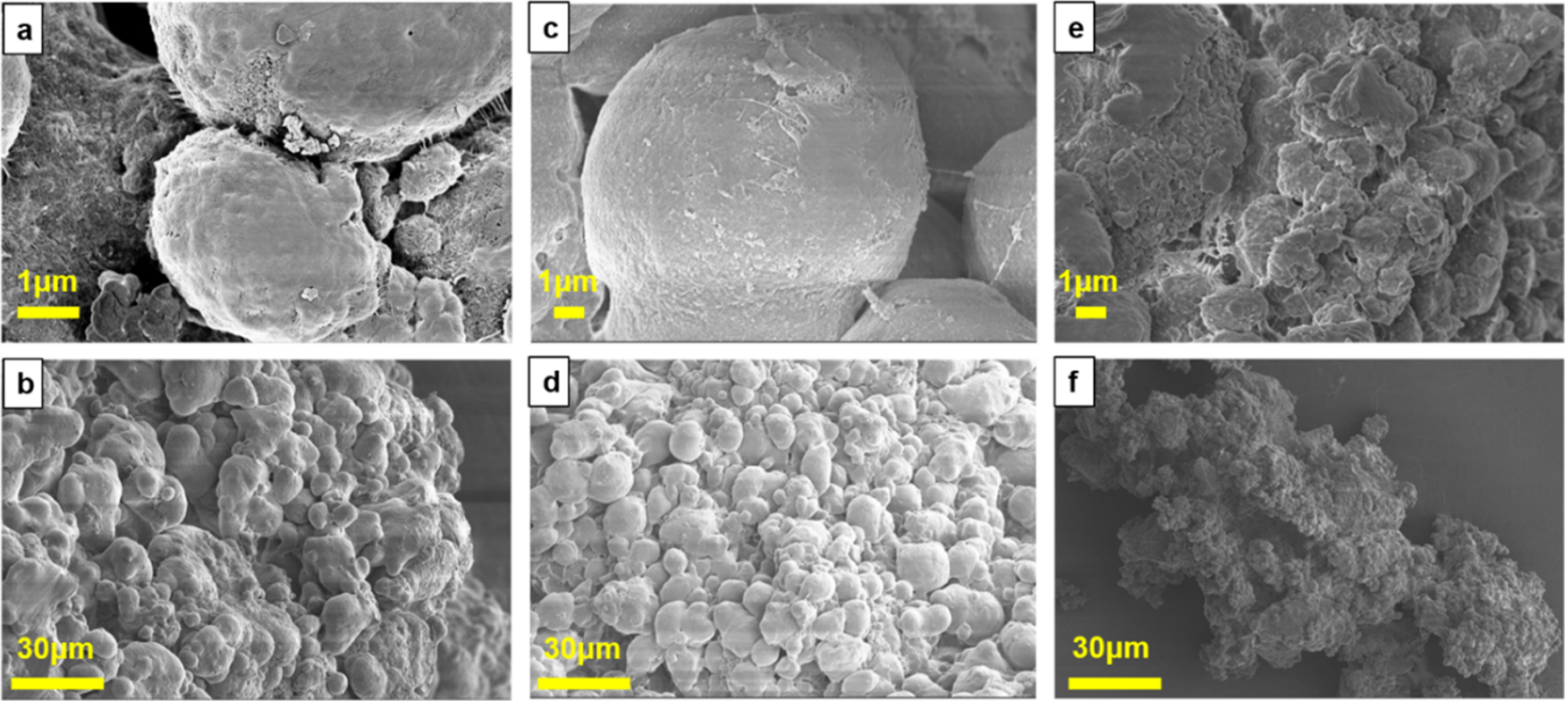
Scanning electron microscopy (SEM) images of polyethylene (PE) products produced from the metal–organic framework (MOF)-supported metallocene catalyst materials: (a, b) MOF-5/MAO/Zr, (c, d) IRMOF-3/MAO/Zr, and (e, f) ZIF-8/MAO/Zr.

The X-ray diffraction (XRD) patterns of the polyethylene (PE) products made are displayed in Figure 7b. The results exhibit the typical features of either HDPE or UHMWPE and agree well with literature data.^54,55^ Two typical {110} and {200} reflections of orthorhombic polyethylene at ∼2θ = 21.5 and ∼23.9° and a {010} reflection of monoclinic polyethylene at ∼2θ = 19.5° can be observed. From the previous discussion in Section 3.1, it is inferred that MAO and Zr are mainly present inside the MOF-5 and IRMOF-3 materials and mainly deposited externally on the ZIF-8 material. MOF/MAO/Zr catalyst materials exhibit pore size distributions at ∼1–2 nm, which are fairly enough for the access of ethylene (with a kinetic diameter of 4.16 Å). Moreover, the fragile frameworks of these MOFs and high ethylene polymerization activities of the MOF-supported metallocenes also indicated that the efficient fragmentation of MOF supports and continuous access of ethylene to Zr active sites with growing oligomers appended, which can be further supported by the SEM images. From the SEM images of the catalysts (Figure 3) and PE products (Figure 8), the polymer products are expanded from the supported catalysts. Plenty of spherical PE particles are observed for the polymer produced from MOF-5- and IRMOF-3-supported catalysts, indicating the efficient fragmentation of the MOF-5 and IRMOF-3 support and continuous growth of PE. The polymer from the ZIF-8 support shows smaller and rougher particles yet still is much larger than the catalyst particles. The significant difference in Zn content between the PE product and the pristine MOFs also indicates the high fragmentation degree of the MOF supports (Table S1).

## 4 Conclusions

Three distinct Zn-based metal–organic frameworks (MOFs), namely, MOF-5, IRMOF-3, and ZIF-8, with different linkers have been used as support materials for metallocene complexes, active for the catalytic polymerization of ethylene. In this manner, the effect of pore size, functional groups of the linker molecule, and the surface groups of MOFs on the ethylene polymerization activity and the related polyethylene properties could be studied. The MOF-5 material with large pore size, no functional groups on its linker, and less surface groups exhibited an Al/Zr ratio of 23 for MOF-5/MAO/Zr and the highest ethylene polymerization activity (i.e., 373 kg PE (mol Zr)^−1^ h^–1^) among these three MOF materials, indicating a low amount of MAO material required for the activation of the zirconocene complexes. For the IRMOF-3 material, the presence of amino groups on its linker (which can act as Brønsted acid sites) required higher amounts of MAO material to scavenge these poisonous functional groups, resulting in a higher Al/Zr ratio of 91 and an ethylene polymerization activity of 293 kg of PE (mol Zr)^−1^ h^–1^. The small aperture and complex and diverse surface groups of the ZIF-8 material resulted in the need for more MAO material, and this MAO was mainly grafted on its surface, resulting in the highest Al/Zr ratio of 101 and the lowest activity of 269 kg PE (mol Zr)^−1^ h^–1^. These findings highlight the influence of functional groups and surface groups of MOF material as support for metallocene complexes, active in olefin polymerization. Moreover, rather than a single-site behavior for the silica-supported metallocene materials, a multisite behavior was observed for the MOF-supported metallocene materials. The three MOF-supported metallocene catalyst materials produced polyethylene with a range of properties, even including the formation of ultrahigh molecular weight polyethylene (i.e., *M*_W_ = 5.34 × 10^6^), indicating the high tunability of MOF materials as support for anchoring metallocene complexes.

## Supporting Information Available

Chemicals, MOF synthesis, crystallinity calculations, Zn content of MOFs and PE products, GPC details, molecular weight and molecular distribution calculations of polyethylene, and Fourier transform infrared (FT-IR) spectra upon CO adsorption from 0 to 5 mbar (PDF)
