# Supplementary material for: Ethylene Polymerization over Metal–Organic Framework-Supported Zirconocene Complexes
Source: ACS Catal. 2024 May 29;14(11):9093–103. doi: 10.1021/acscatal.4c01061 (PMC11165446; doi:10.1021/acscatal.4c01061)
Supplement: Supplementary file 1 — cs4c01061_si_001.pdf [file cs4c01061_si_001.pdf]

Supporting Information

Ethylene Polymerization over Metal-Organic Frameworks-Supported Zirconocene Complexes

Yaqi Wu,[1, 2] Joren M. Dorresteijn,[1] and Bert M. Weckhuysen[1,]*

[1] Inorganic Chemistry and Catalysis group, Institute for Sustainable and Circular Chemistry and Debye Institute for Nanomaterials Science, Utrecht University, Universiteitsweg 99, 3584 CG Utrecht, the Netherlands

[2] Hydrogen Energy Utilization and Energy Storage Technology Laboratory, Ningbo Institute of Materials Technology and Engineering, Chinese Academy of Sciences, Ningbo, Zhejiang, 315201 P. R. China

Corresponding Author

E-mail address: b.m.weckhuysen@uu.nl

1. Addtional experimental section

1.1 Chemicals

$Zn(NO_3)_2$•$6H_2O$ (98%, Sigma-Aldrich), terephthalic acid (98%, Sigma-Aldrich), 2-aminoterephthalic acid (99%, Sigma-Aldrich), 2-methylimidazole (Sigma–Aldrich, 99%), *N,N*-dimethylformamide (Extra pure, Fisher), methanol (Acros, >=99.9%), heptane (anhydrous, over molecular sieves, Ar, ChemSeal), Toluene (99.85%, Fisher), n-Pentane (99+%, Thermo Scientific), dichlomethane (>=99.9%, VMR chemicals), Cp_2ZrMe_2 (bis(cyclopentadienyl)dimethyl-zirconium (IV); 97%, Sigma Aldrich), MAO (13 wt% Al in toluene, Akzo-Nobel), triethylaluminum (25 wt% in toluene, Sigma-Aldrich), ethylene (C_2H_4 4.0, Linde).

1.2 MOF synthesis

MOF-5,[1] IRMOF-3[2] and ZIF-8[3] were synthesized according to the reported procedures with some modifications. MOF-5: 0.446 g $Zn(NO_3)_2$•$6H_2O$, 0.083 g terephthalic acid and 10 mL *N,N*-dimethylformamide (DMF) were mixed together and then heated to 150 ˚C for 6 h. IRMOF-3: 0.328 g $Zn(NO_3)_2$•$6H_2O$, 0.075 g 2-aminoterephthalic acid and 10 mL DMF were mixed together and then heated to 150 ˚C for 6 h. The as-prepared MOF-5 and IRMOF-3 were washed with DMF

three times and the soaked in chloroform for 3 days (thereby changing the solvent at least 3 times).

ZIF-8: A solution of 258 g $Zn(NO_3)_2$•$6H_2O$ in 100 mL methanol was added to a solution of 263 g 2-methylimidazole in 100 mL methanol under stirring and kept stirring for 1 h. Then the solution was aged at room temperature for 24 h. The as-prepared ZIF-8 was washed with methanol for 3 times. MOF-5, IRMOF-3 and ZIF-8 were calcinated at 200 ˚C overnight under N_2 flow before stored in glovebox and further use.

1.3 Characterizations

The crystallinity of the pristine MOFs, MOF/MAO, and MOF/MAO/Zr samples are calculated based on their XRD patterns according to the method reported by Patience et al.[4] The equation can be expressed as follows:

$$C = 100\frac{\Sigma N_{net}}{\Sigma N_{tot} - \Sigma N_{scat}}$$

where N_{net} is the total intensities in the diffraction pattern, N_{tot} is the sum of all the measured intensities, N_{scat} is the intensities of air scatter. The air scatter is obtained by measuring a zero background holder.

The molecular weight (M_n (number average molecular weight), M_w (weight average molecular weight) and molecular weight distributions D (M_w/M_n), and D' (M_z/M_w) were determined by size exclusion chromatography (SEC) and in particular by IR-detected gel permeation chromatography (GPC) at high temperature (145 °C). Briefly, a GPC-IR5MCT from Polymer Char was used: 8 mg polymer sample was dissolved at 160 °C in 8 mL of trichlorobenzene stabilized with 1000 ppm by weight of butylhydroxytoluene (BHT) for 1 hour (h). Injection volume: about 400 μl, automatic sample preparation and injection temperature: 160 °C. Column temperature: 145 °C. Detector temperature: 160 °C. Column set: two Shodex AT-806MS (Showa Denko) and one Styragel HT6E (Waters), columns were used with a flow rate of 1 mL/min. Detector: Infrared detector (2800-3000 cm^{-1}) to collect all C-H bonds and two narrow band filters tuned to the absorption region assigned to CH_3 and CH_2 groups. Calibration: narrow standards of polystyrene (PS) (commercially available). Calculation of molecular weight M_i of each fraction i of eluted polymer is based on the

Mark-Houwink relation ($\log_{10}(M_{PE})$ = 0.965909 x $\log10(M_{PS})$ – 0.28264) (cut off on the low molecular weight end at M_{PE} = 1000).

The molecular weight averages used in establishing molecular weight/property relationships are the number average (M_n), weight average (M_w) and z average (M_z) molecular weight. These averages are defined by the following expressions and are determined form the calculated M_i:

$$M_n = \frac{\sum_i N_i M_i}{\sum_i N_i} = \frac{\sum_i W_i}{\sum_i W_i / M_i} = \frac{\sum_i h_i}{\sum_i h_i / M_i}$$

$$M_w = \frac{\sum_i N_i M_i^2}{\sum_i N_i M_i} = \frac{\sum_i W_i M_i}{\sum_i W_i} = \frac{\sum_i h_i M_i}{\sum_i h_i}$$

$$M_z = \frac{\sum_i N_i M_i^3}{\sum_i N_i M_i^2} = \frac{\sum_i W_i M_i^2}{\sum_i W_i M_i} = \frac{\sum_i h_i M_i^2}{\sum_i h_i M_i}$$

Here N_i and W_i are the number and weight, respectively, of molecules having molecular weight M_i. The third representation in each case (farthest right) defines how one obtains these averages from SEC chromatograms. h_i is the height (from baseline) of the SEC curve at the i_{th} elution fraction and M_i is the molecular weight of species eluting at this increment.

2. Additional Data

Table S1. Zn content of MOFs and PE products as obtained by Inductively Coupled Plasma-Optical Emission Spectroscopy (ICP-OES).

Zn content (wt%)	MOFs	PE products
MOF-5	36.5	0.035
IRMOF-3	34.7	3
ZIF-8	25.5	0.029

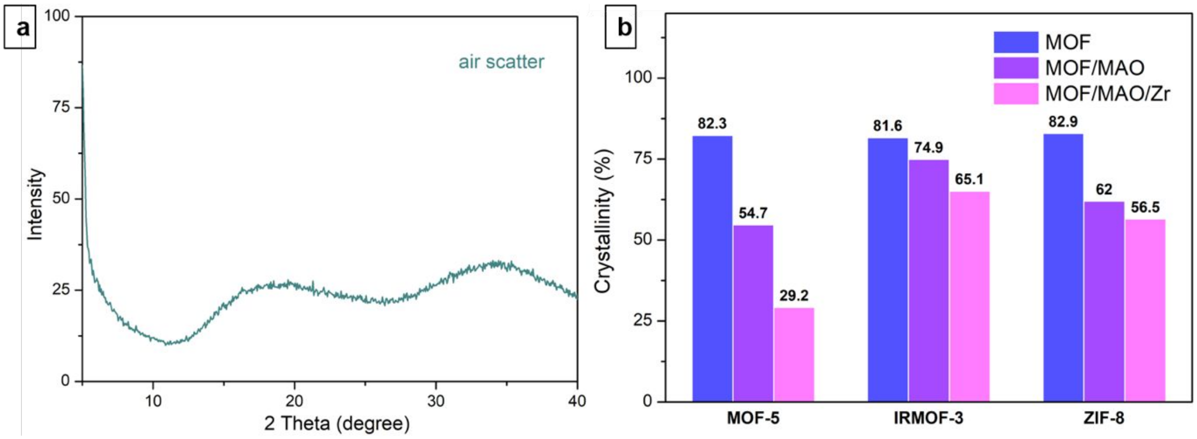

Figure S1. X-ray diffraction (XRD) patterns of blank sample holder and histograms of crystallinity data of pristine, MAO-loaded and Zr/MAO-loaded MOF-5, IRMOF-3 and ZIF-8.

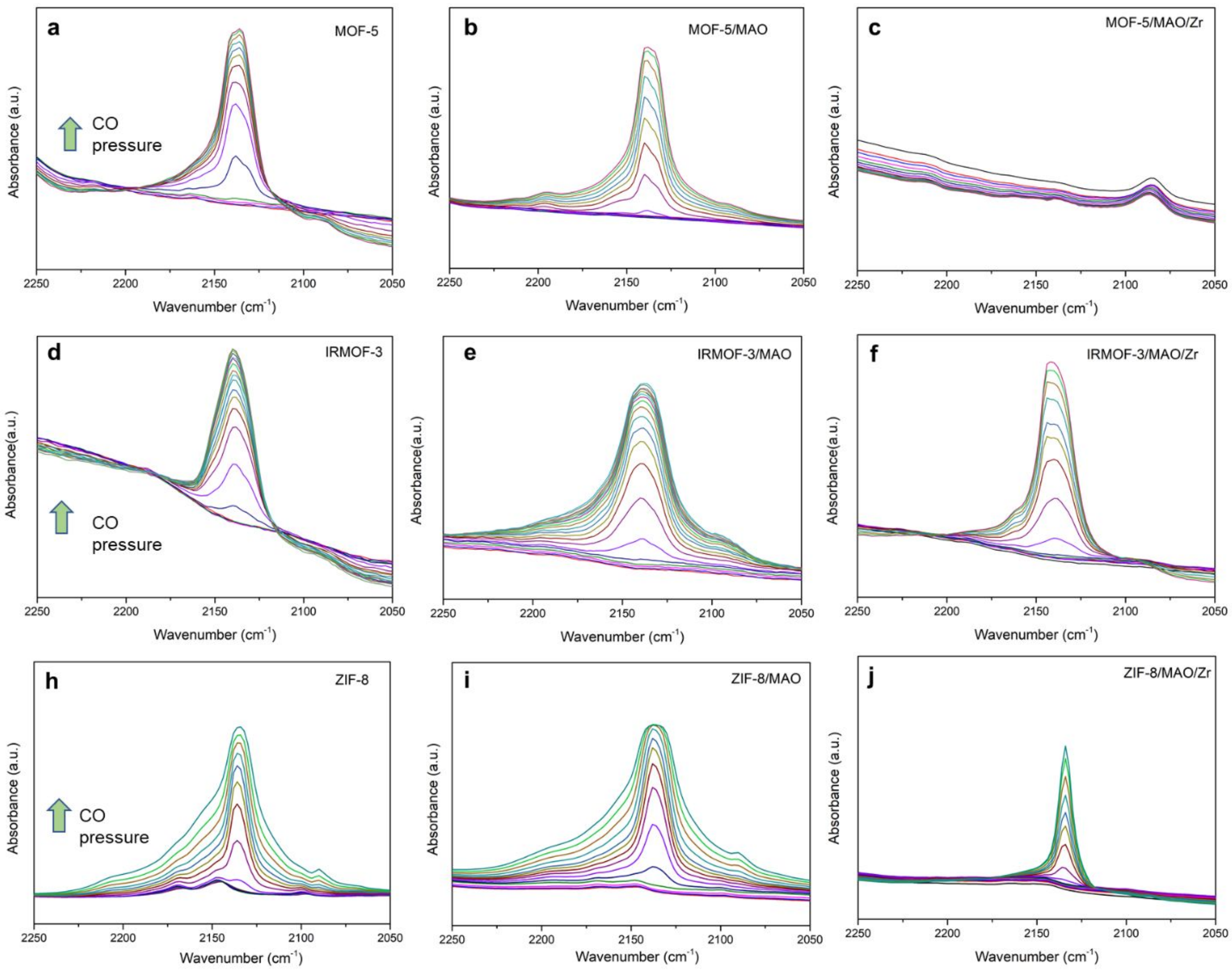

Fig. S2 Fourier Transform-Infrared (FT-IR) spectra for (a) MOF-5, (b) IRMOF-3 and (c) ZIF-8 before and after metal aluminoxane (MAO) impregnation and Zr loading upon CO adsorption at -188 ˚C and from 0-5 mbar.

References

(1) Chen, B.; Wang, X.; Zhang, Q.; Xi, X.; Cai, J.; Qi, H.; Shi, S.; Wang, J.; Yuan, D.; Fang, M. Synthesis and characterization of the interpenetrated MOF-5. *J. Mater. Chem.* 2010, *20* (18), 3758-3767.

(2) Tanabe, K. K.; Wang, Z.; Cohen, S. M. Systematic Functionalization of a Metal−Organic Framework via a Postsynthetic Modification Approach. *J. Am. Chem. Soc.* 2008, *130* (26), 8508-8517.

(3) Li, H.; Fu, D.; Zhang, X.-M.; Han, G.; Zhang, F. Facile Preparation of Varisized ZIF-8 and ZIF-8/Polypyrrole Composites for Flexible Solid-State Supercapacitor. *ChemistrySelect* 2017, *2* (25), 7530-7534.

(4) Khan, H.; Yerramilli, A. S.; D'Oliveira, A.; Alford, T. L.; Boffito, D. C.; Patience, G. S. Experimental methods in chemical engineering: X-ray diffraction spectroscopy—XRD. *The Can. J. Chem. Engin.* 2020, *98* (6), 1255-1266.
